# Does the low-density lipoprotein cholesterol play a key role in predicting metabolic syndrome in the Iranian adult population?

**DOI:** 10.22088/cjim.8.4.289

**Published:** 2017

**Authors:** Karimollah Hajian-Tilaki, Behzad Heidari, Arefeh Hajian-Tilaki, Alireza Firouzjahi, Afsaneh Bakhtiari

**Affiliations:** 1 Social Determinant Health Research Center, Health Research Institute, Babol University of Medical Sciences, Babol, Iran.; 2 Mobility Impairment Research Center, Health Research Institute, Babol University of Medical Sciences, Babol, Iran.; 3 Department of Orthodontics, Guilan University of Medical Sciences, Rasht, Iran.; 4 Cancer Research Center, Health Research Institute, Babol University of Medical Sciences, Babol, Iran.; 5 Fatemeh Zahra Infertility and Reproductive Health Research Center, Health Research Institute, Babol University of Medical Sciences, Babol, Iran.

**Keywords:** Low density lipoprotein cholesterol, Metabolic syndrome, Iranian adults

## Abstract

**Background:**

The low density lipoprotein cholestrol (LDL-C) has an important role in the pathogenesis of cardiovascular disease but its association and predictive accuracy with metabolic syndrome (MetS) remains controversial. The objective of this study was to investigate the association and predictive ability of LDL-C with MetS.

**Methods:**

We analyzed the data from a population-based cross-sectional study conducted on representative samples of an Iranian adult population. The demographic data, anthropometric measures and the lipid profiles were measured with standard methods, and MetS was diagnosed by ATP III criteria. Logistic regression model and ROC analysis were used to estimate the predictive accuracy of LDL-C and its association with MetS.

**Results:**

The mean age (±SD) of participants with and without MetS was 47.6±12.5 years and 39.1±12.9 years, respectively (p=0.001). All anthropometric measures (body mass index, waist circumference, waist to hip ratio, waist to height ratio), systolic blood pressure, total cholesterol, triglycerides and fasting blood glucose were significantly higher in MetS, but a significantly higher difference in LDL-C was observed only in women. Accuracy of LDL-C in predicting MetS for men and women was 0.48 (95% CI: 0.43-0.54) and 0.55 (95% CI: 0.51-0.60), respectively. The unadjusted and adjusted odds ratios of different quartiles of LDL-C compared with 1^st^ quartile did not reach to a significant level.

**Conclusion:**

Serum LDL-C level is not significantly associated with MetS but exhibits a weak ability in predicting MetS in women.

**M**etabolic syndrome (MetS) constitutes a clustering of cardiovascular risk factors including high blood pressure, increased fasting blood sugar (FBS), high serum triglycerides level (TG), low high density lipoprotein(HDL), and abdominal obesity (1, 2). It is a major risk factor for diabetes and cardiovascular event and stroke (3). Individuals with MetS have at least three times greater risk of cardiovascular complications and two times higher risk of death as compared with those without MetS (4-7). It is estimated that more than a quarter of adult population in the United States suffer from MetS (8), and its prevalence has increased dramatically in Iranian population during the two recent decades as well, and according to ethnic, geographic regions and socio-demographic characteristics, the prevalence of MetS in Iran ranges from 10- 60% (9-14). In particular, obesity and MetS are highly prevalent in geographic regions of northern Iran (15-17). Obesity, especially abdominal obesity constitutes the main components of MetS (1, 2). It has been clearly established that there is a link between obesity/central obesity and hypertension as well as other cardiometabolic risk factors in particular dyslipidemia (18-21). 

Although low density lipoprotein cholesterol (LDL-C) is not a components of MetS, nevertheless, both hyperlioidemia and MetS are associated with abdominal obesity and general adiposity. It has been clearly established that LDL-C is an important risk factor of cardiovascular diseases (18-20), however, data regarding the association between LDL-C and MetS are scarce and the results of studies are controversial (21, 22). 

The information in this context is very important because both LDL-C and MetS are factors for future development of cardiovascular complications which are clinically emphasized to be recognized for preventive measures. Identification of subjects with MetS based on serum LDL-C measurement, which is routinely determined in clinical practice, provides an opportunity to treat both conditions simultaneously. 

Since the mainstay of treatment for dyslipidemia is based on lifestyle modifications including weight reduction and correction of abdominal obesity improves MetS and exerts additional benefits in reducing the risk of diabetes as well as cardiovascular complications. Thus, the objective of this study was to determine the ability of LDL-C in predicting MetS, independent of pre-existing components in a sample of north’s Iranian adult population.

## Methods

**Design and study subjects: **We analyzed the data of the population-based cross-sectional of Babol Lipid and Glucose study which was conducted with 1000 representative samples of urban community-dwelling individuals aged 20-70 years from April to end of December in 2012. The sampling procedure and the criteria for recruitment were described in details elsewhere (17). In brief, a random sample of 25 clusters was selected and around the center of each cluster, about 40 subjects were recruited to participate in the study. Individuals with history of atherosclerotic diseases such as myocardial infarction and cerebrovascular accidents (CVA), dementia, current cancer under radio-chemotherapy and pregnancy were excluded. All participants gave a written consent prior to participation. The study protocol was approved by the Ethics Committee of Babol University of Medical Sciences.

**Data Collection: **In a household survey, first the demographic data were collected during a face-to-face interview by trained nurses. Then, the clinical examination and anthropometric assessment were performed. Systolic, diastolic blood pressure, weight, height, waist (WC) and hip circumferences (HC) were measured with standard methods. From these anthropometric measures, body mass index (BMI), waist to hip ratio (WHR), waist to height ratio (WHtR) and abdominal volume index (AVI) were calculated. All participants were invited to central lab of the Ayatollah Rouhani Hospital with overnight fasting of 10-12 hours. The blood samples were provided and the biochemical parameters such as total cholesterol (TC), TG, LDL-C, HDL-C and FBS were measured enzymatically by automated analyzer. The MetS was confirmed by ATP III criteria. In this regard, the presence of at least three out of five components were considered as having MetS.

**Statistical Analysis: **The data were analyzed using SPSS software Version 18 In bivariate data analysis, we used the independent t-test for quantitative and the chi-square test for categorical data. The normality of data was examined by Kolmogrov –Smirnov test. The LDL-C was categorized based on its quartile with respect to gender. The three indicator variables were defined and the reference group was the 1^st^ quartile. Multiple logistic regression analysis was used to estimate the unadjusted and adjusted odds ratio (OR) and its 95% confidence interval (CI). 

Besides age-and-sex adjusted OR was estimated and additional adjustment was performed with pre-existing components of MetS. The receiver operator characteristic (ROC) curve analysis was used to calculate the diagnostic accuracy as defined by the area under the curve (AUC) and its 95% CI. The Pearson test was used to determine the correlation between the components of cardiometabolic risk factors. A p-value less than 0.05 as considered as the significant level. 

## Results

The consecutive mean (±SD) age of participants with and without MetS were 47.6±12.5 and 39.1±12.9 years, respectively (p=0.001). As shown in Table 1, in both genders, all anthropometric measures, except height, were significantly higher in MetS group as compared with those without MetS. In addition, systolic blood pressure, total cholesterol, triglycerides, HDL-C and FBS were significantly higher in both women and men. Nonetheless, LDL-C was significantly higher only in women but not in men with MetS (table 2). 

**Table 1:** The mean (SD) of cardiometabolic risk factors of participants with and without MetS

**Cardiometabolic** ** risk factors**	** Male (mean±SD)**			** Female (mean±SD)**		
	**MetS**	**Not MetS**	**p-value**	**MetS**	**Not MetS**	**p-value**
Age (year)	47.6±14.3	41.2±13.8	0.001	47.5±11.2	36.9±11.6	0..001
Weight (kg)	83.4±13.3	74.8±13.0	0.001	78.4±12.8	66.9±12.1	0.001
Height (cm)	171.6±8.3	171.9±7.7	0.76	158.4±7.3	159.7±6.8	0.036
BMI (kg/m2)	28.3±3.9	25.4±4.9	0.001	31.3±5.4	26.3±4.8	0.001
WC (cm)	102 .0±15.2	89.3±12.4	0.001	99.0±713.5	84.2±12.1	0.001
HC (cm)	106.5±11.3	99.7±12.5	0.001	114.0±11.9	104.6±13.2	0.001
WHR	0.94±0.10	0.89±0.08	0.001	0.87±0.08	0.81±0.08	0.001
WHtR	0.59±0.09	0.52±0.07	0.001	0.63 ±0.09	0.52±0.08	0.001
AVI	20.91±6.8	16.4±4.8	0.001	20.50±6.3	14.8±4.2	0.001
Diastolic BP (mm Hg)	87.9±12.5	79.5±12.1	0.001	87.6±8.16	757±11.6	0.001
Systolic BP (mm Hg)	135.4±15.1	124.1±14.4	0.001	131.9±20.3	117.4±15.0	0.001
Total cholesterol (mgr/dl)	201.6±51.0	185.9±58.7	0.001	211.5±43.7	187.2±40.6	0.001
TG (mg/dl)	256.4±151.6	151.3±115.8	0.001	208.5±136.3	110.2±64.2	0.001
LDL-C	118.8±51.1	117.5±38.3	0.75	133.2±41.9	126.1±36.5	0.037
HDL-C	32.9±6.7	37.8±10.7	0.001	37.4±12.9	39.8±11.2	0.02
FBS	125.2±43.8	98.7±22.9	0.001	125.5±54.2	94.8±16.9	0.001

BMI body mass index, WC, waist circumference, HC hip circumference, WHR waist to hip ratio, WHtR waist to height ratio, AVI abdominal volume index, BP blood pressure, TG triglycerides, LDL-C low density lipoprotein cholesterol, HDL-C high density lipoprotein cholesterol, FBS fasting blood sugar

**Table 2:** The Pearson correlation matrix between cardiometabolic risk factors and P-value

	**CHL**	**TG**	**LDL-C**	**HDL-C**	**FBS**	**Diastolic BP**	**Systolic BP**	**WC**	**BMI**
**CHL Correlation** **P-value**	1	0.32 0.001	0.76 0.001	0.11 0.001	0.15 0.001	0.14 0.001	0.14 0.001	0.17 0.001	0.18
**TG Correlation** **P-value**		1	-0.16 0.001	-0.13 0.001	0.29 0.001	0.11 0.001	0.14 0.001	0.21 0.001	0.14 0.001
**LDL-C Correlation** **P-value**			1	0.02 0.44	0.02 0.44	0.09 0.004	0.08 0.01	0.08 0.01	0.13 0.001
**HDL-C Correlation** **P-value**				1	-0.05 0.11	0.06 0.05	0.07 0.03	0.02 0.45	0.03 0.32
**FBS Correlation** **P-value**					1	0.15 0.001	0.19 0.001	0.02 0.45	0.07 0.03
**Diastolic BP Correlation** **P-value**						1	0.64 0.001	0.23 0.001	0.19 0.001
**Systolic BP Correlation** **P-value**							1	0.27 0.001	0.21 0.001
**WC Correlation** **P-value**								1	0.62 0.001
**BMI Correlation** **P-value**									1

CHL cholesterol, TG triglycerides, LDL-C low density lipoprotein cholesterol, HDL-C high density lipoprotein cholesterol, FBS fasting blood sugar, BP blood pressure, WC waist circumference, BMI body mass index

**Figure 1:**
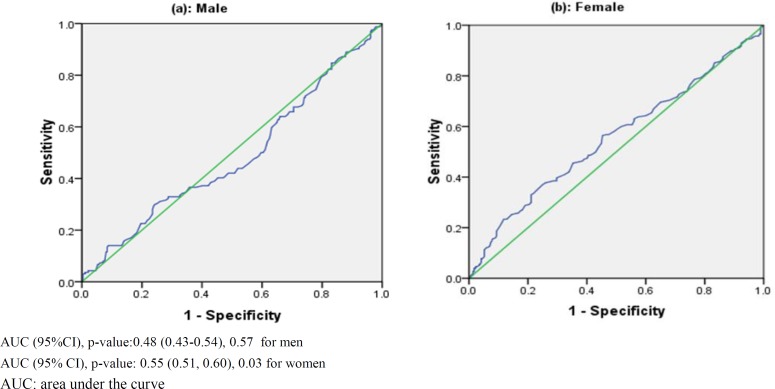
ROC curves and accuracy index of LDL-C for predicting of in men (panel a) and women (panel b

Among the components of the MetS, LDL-C was positively correlated with systolic blood pressure (r=0.08, P=0.01), BMI (r=0.13, P=0.001), and WC (r=0.08, P=0.02). Apparently there was a strong correlation between LDL-C and total cholesterol (r=0.76, P=0.001) but there was no significant correlation between LDL-C with HDL-C and FBS. Surprisingly, the correlation between LDL-C and TG was negative. Regarding the data presented in figure 1, the LDL-C has no diagnostic ability in predicting MetS among men (AUC=0.48, 95% CI: 0.43-0.54), but there is a significant and low predicting ability for MetS in women (AUC=0.55, 95%CI: 0.51-0.60). 

Nonetheless, the result of multiple logistic regression analysis (table 3) showed that the unadjusted and adjusted odds ratios (adjusted through age and gender, and additional adjustment for preexisting component of the MetS) of the 4^th^, 3^rd^ and 2nd quartiles of LDL-C versus the 1^st^ quartile did not indicate a statistically significant association.

**Table 3:** The unadjusted and adjusted odds ratio (OR) and its 95% confidence interval (CI) of different quartiles of HDL-C using multiple logistic regression model

**LDL-C**	**Unadjusted ** **OR (95%CI)**	**P-value**	Adjusted* **OR(95% CI)**	**P-value**	**Adjusted** ** **OR(95%CI)**	**P-value**
1^st^ Quartile 2^nd^ Quartile 3^rd^ Quartile 4rth Quartile	1 0.92 (0.65, 1.31) 0.80 (0.56, 1.14) 1.25 (0.88, 1.78)	0.09 0.65 0.21 0.21	1 (-) 0.92 (0.63, 1.34) 0.65 (0.44, 0.95) 0.93 (0.63, 1.36)	0.12 0.68 0.03 0.17	1 (-) 1.06 (0.63, 1.80) 0.79 (0.46, 1.33) 1.02 (0.60,1.77)	0.70 0.82 0.41 0.96

* Adjusted by age and sex

** Full adjusted by age, gender, BMI, and all pre-existing components of MetS. LDL-C low density lipoprotein cholesterol, OR odds ratio

## Discussion

The findings of this study demonstrated significant differences regarding all demographic and biochemical factors of coronary artery disease between subjects with and without MetS. The values of BMI, WC, WHR, WHtR, AVI, FBS, TC, TG, systolic and diastolic blood pressure in individuals with MetS were higher and the level of HDL-C was lower in both women and men. Whereas, the level of LDL-C was significantly higher only in female with MetS but not in males. Based on the value of the area under the ROC curve, LDL-C yielded a weak ability in prediction of MetS only in women. Both LDL-C and MetS are prevalent in the general population, and are associated with increased risk of cardiovascular mortalities and CVS (23, 24). Many chronic medical conditions, like obesity, diabetes, dyslipidemia and hypertension are related with MetS. Hence, coexistence of these conditions with MetS poses these subjects at greater risk of cardiovascular events. The cardiovascular risk in MetS and LDL-C is comparable, and thus, coexistence of two conditions yields the risk of cardiovascular by sum of the two risk factors (21). 

In a study of patients with MetS the patients at very higher risk of cardiovascular death had greater LDL-C and non-HDL-C levels than those with medium or high risk of cardiovascular death (25). So, the reduction of LDL-C in MetS confers the beneficial effect against cardiovascular diseases. In practice, reduction of LDC-C to target level by proper treatment reduces the risk but does not prevent subsequent attacks. This may be related to the presence of other risk factors such as MetS, which may reduce the effectiveness of treatment (26). High level of LDL-C and low level of HDL-C are risk factors of coronary artery disease. Patients with MetS are at greater risk of dyslipidemia(14). 

Low level of HDL-C is a component of MetS, but data regarding LDL-C and MetS are lacking. A study of Japanese population showed a relationship between LDL-C and MetS; in this study LDL-C predicted development of MetS, independent of BMI, or MetS components (22). In another follow-up study of 1702 participants without MetS at baseline, individuals who developed MetS, had significantly higher LDL-C, apolipoprotein B, C-reactive protein and lower HDL-C (27). In another study, by Ying et al, the LDL- C independently predicted the MetS (28). Nonetheless, in a 7-year follow -up study of Iranian cohort comprised of first degree relatives of diabetic patients, LDL-C did not yield a predictive ability for MetS independent of age or the pre-existing components of the MetS (23).

Similar to most components of Mets, LDL-C is also associated with insulin resistance (29). Similarly, the ratio of TG to HDL-C is an indicator of insulin resistance. This was shown in a longitudinal study of Iranian cohort followed for a median duration of 6.5 years (30). In reality, the risk of cardiovascular disease is determined by the direct measurement of LDL and HDL particles. The magnitude of cholesterol especially LDL per particle varies across different persons. In patients with low HDL, the risk of cardiovascular disease may be related to unrecognized excess of LDL particles (31).

The LDL-C and size are often accompanied with low HDL-C and high TG levels (26). TG/HDL-C ratio is an indicator of MetS and insulin resistance and development cardiovascular events (32). Small dense LDL with elevated TG and low HDL-C concentrations constitute the atherogenic lipoprotein phenotype which is a feature of diabetes type 2 and MetS. Small dens LDL assessment may be helpful in predicting cardiovascular risk in MetS (32). 

The results of this study showed a weak predictive ability of LDL-C for MetS in women but not in men, which is partly in agreement with Tehrani et al. who have shown no association between LDL-C and coronary heart disease or cardiovascular disease in the MetS group (33). 

The present study has limitations. The cross-sectional nature of the study does not indicate causality. Additionally, we did not exclude subjects who are on treatment of antihyperlipidemic agents as a possible confounder. Nevertheless, the distribution of subjects who were taking treatment is expected to be similar across the comparison groups. As a conquence, the results are subjected to be less confounded. 

Yet, the strength is a population-based with standard sampling technique and large sample size, using appropriate criteria for definition of MetS and standard methods of data collection and analysis techniques. In addition, the study population was recruited among the general population with similarities in many characteristics such as lifestyle, ethnicity, and socio-demographic features. Thus, the study population can be considered as representative of the general population.

In conclusion although LDL-C correlates with a number of MetS components, but the level of LDL-C in subjects with and without MetS was not significantly different and as a result the LDL-C did not yield predictive ability in detecting MetS except a weak predictive ability in women. At any rate, this issue needs to be confirmed further in a prospective longitudinal study. 

## References

[1] Grundy SM, Brewer HB, Cleeman JI (2004). Definition of metabolic syndrome: report of the National Heart, Lung and Blood Institute/American Heart Association Conference on scientific issues related to definition. Circulation.

[2] Executive Summary of The Third Report of The National Cholesterol Education Program (NCEP) expert panel on detection (2001). evaluation and Treatment of high blood cholesterol in adults (Adult Treatment Panel III). JAMA.

[3] Lloyd–Jones D, Adams R, Carnethom MD (2009). Heart disease and stroke statistics-2009 update: a report from the American Heart Association Statistics Committee and Stroke Statistics Subcommittee. Circulation.

[4] Morttillo S, Filion KB, Genest J (2010). The metabolic syndrome and cardiovascular risk: a systematic review and meta analysis. Am J Coll Cardiol.

[5] Isomaa B, Almgren P, Tuomi T (2001). Cardiovascular mortality and morbidity associated with metabolic syndrome. Diabetes Care.

[6] Church TS, Thompson AM, Katzmarzyk PT (2009). Metabolic syndrome and diabetes, alone and in combination as predictors of cardiovascular disease mortality among men. Diabetes Care.

[7] Ford ES (2004). The metabolic syndrome and mortality from cardiovascular disease and all causes: findings from the National Health and Nutrition Examination Survey II Mortality Study. Atherosclerosis.

[8] Fakhrzadeh H, Ebrahimpour P, Pourebrahim R, Heshmat R, Larijani B (2006). Metabolic syndrome and its associated risk factors in healthy adults: A population-based study in Iran. Metab Syndr Relat Disord.

[9] Esteghamati A, Zandieh A, Khalilzadeh O, Meysamie A, Ashraf H (2010). Clustering of metabolicsyndrome components in a MiddleEasterndiabetic and non-diabetic population. Diabeto Metab Syndr.

[10] Yousefzadeh G, Sheikhvatan M (2015). Age and gender differences in the clustering of metabolic syndrome combinations: A prospective cohort research from the Kerman Coronary Artery Disease Risk Study (KERCADRS). Diabetes Metab Syndr.

[11] Azizi F, Salehi P, Etemadi A, Zahedi-Asl S (2003). Prevalence of metabolic syndrome in urban population: Tehran Lipid and Glucose Study. Diabetes Res Clin Pract.

[12] Sarrafzadegan N, Kelishadi R, Baghaei A (2008). Metabolic syndrome: An emerging public health problem in Iranian women: Isfahan Healthy Heart Program. Int J Cadiol.

[13] Ebrahimi M, Kazemi-Bajestani SM, Ghayour-Mobarhan M, Ferns GA (2011). Coronary artery disease and its risk factors status in Iran: a review. Iran Red Crescent Med.

[14] Hajian-Tilaki K (2015). Metabolic syndrome and the associated risk factors in Iranian adults: a systematic review. Caspian J Intern Med.

[15] Hajian-Tilaki K, Heidari B (2007). Prevalence of obesity, central obesity and the associated factors in urban population aged 20-7years, in the north of Iran: a population-based study and regression approach. Obs Rev.

[16] Hajian-Tilaki K, Jalali F (2007). Changing patterns of cardiovascular risk factors in hospitalized patients with acute myocardial infarction in Babol, Iran. Kuwait Med J.

[17] Hajian-Tilaki K, Heidari B, Firozjahi A (2014). Prevalence of metabolic syndrome and the associated socio-demographic characteristics and physical activity in urban population of Iranian adults: a population-based study. Diabetes Metab Syndr.

[18] Smith SC Jr (2007). Multiple risk factors for cardiovascular disease and diabetes mellitus. Am J Med.

[19] Rouvre M, Vol S, Gusto G (2011). Low density lipoprotein cholesterol: prevalence and associated risk-factors in a large French population. Ann Epidemiol.

[20] Connelly PW, Petrasovits A, Stachenko S (1999). Prevalence of high plasma triglyceride combined with low HDL-C levels and its association with smoking, hypertension, obesity, diabetes, sedentariness and LDL-C levels in the Canadian population. Canadian Heart Health Surveys Research Group. Can J Cardiol.

[21] Jeppesen J, Hansen WH, Rasmussen S (2006). Metabolic syndrome,low density lipoprotein cholesterol and risk of cardiovascular disease: A population based study. Atherosclerosis.

[22] Oda E (2013). Low density lipoprotein cholesterol is a predictor of metabolic syndrome in a Japanese health screening population. Intern Med.

[23] Janghorbani M, Amini M (2015). Low density lipoprotein cholesterol and metabolic syndrome in an Iranian population. Diabetes Metab Syndr.

[24] Amihăesei IC, Chelaru L (2014). Metabolic syndrome a widespread threatening condition; risk factors, diagnostic criteria, therapeutic options, prevention and controversies: an overview. Rev Med Chir Soc Med Nat Iasi.

[25] Gierach M, Gierach J, Junik R (2016). Evaluation of lipid profiles in patients with metabolic syndrome according to cardiovascular risk calculated on the basis of the SCORE chart. Endokrynol Pol.

[26] Mudd JO, Borlaug BA, Johnston PV (2007). Beyond low-density lipoprotein cholesterol: defining the role of low-density lipoprotein heterogeneity in coronary artery disease. J Am Coll Cardiol.

[27] Onat A, Can G, Çakr H (2015). Sex-specific predictors of metabolic syndrome independent of its components. J Investig Med.

[28] Ying Y, Qian Y, Jiang Y (2012). Association of the lipoprotein B/apolipoprotein A-1ratio and low -density lipoprotein cholesterol with insulin resistance in a Chjnese population with abdominal obesity. Acta Diabetologia.

[29] Makaridze Z, Giorgadze E, Asatiani K (2014). Association of the apolipoprotein b/apolipoprotein a-I ratio, metabolic syndrome components, total cholesterol, and low-density lipoprotein cholesterol with insulin resistance in the population of Georgia. Int J Endocrinol.

[30] Hadaegh F, Khalili D, Ghasemi A (2009). Triglyceride/HDL-cholesterol ratio is an independent predictor for coronary heart disease in population of Iranian men. Nutr Metab Cardiovasc Dis.

[31] Otvos JD, Jeyarajah EJ, Cromwell WC (2002). Measurement issues related to lipoprotein heterogenity. AmJ Cardiol.

[32] Rizzo M, Berneis K (2007). Small, dense low-density-lipoproteins and the metabolic syndrome. Diabetes Metab Res Rev.

[33] Tehrani DM, Zhao Y, Blaha MJ (2016). Discordance of low-density lipoprotein and high-density lipoprotein cholesterol particle versus cholesterol concentration for the prediction of cardiovascular disease in patients with metabolic syndrome and diabetes mellitus (from the Multi-Ethnic Study of Atherosclerosis [MESA]. Am J Cardio.

